# Supplementary material for: Mitochondrial Uncoupling Proteins (UCP1-UCP3) and Adenine Nucleotide Translocase (ANT1) Enhance the Protonophoric Action of 2,4-Dinitrophenol in Mitochondria and Planar Bilayer Membranes
Source: Biomolecules. 2021 Aug 9;11(8):1178. doi: 10.3390/biom11081178 (PMC8392417; doi:10.3390/biom11081178)
Supplement: Supplementary file 1 [file biomolecules-11-01178-s001.zip › biomolecules-1287846-supplementary.pdf]

Supporting information

Mitochondrial uncoupling proteins (UCP1-UCP3) and adenine nucleotide translocase (ANT1) enhance uncoupling action of 2, 4-dinitrophenol in mitochondria and planar lipid bilayers

Kristina Žuna[a], Olga Jovanović[a], Ljudmila S. Khailova[b], Sanja Škulj[c], Zlatko Brkljača[d], Jürgen Kreiter[a], Elena A. Kotova[b], Mario Vazdar[d,e], Yuri N. Antonenko[b,@], Elena E. Pohl[a,@]

[a]Institute of Physiology, Pathophysiology and Biophysics, Department of Biomedical Sciences, University of Veterinary Medicine, Vienna A-1210, Austria
[b]Belozersky Institute of Physico-Chemical Biology, Lomonosov Moscow State University, Leninskie Gory 1/40, 119991 Moscow, Russia
[c] Department of Chemistry, Faculty of Science, University of Zagreb, Horvatovac 102a, 10000 Zagreb, Croatia
[d]Division of Organic Chemistry and Biochemistry, Ruđer Bošković Institute, Bijenička 54, 10000 Zagreb, Croatia
[e]Institute of Organic Chemistry and Biochemistry, Czech Academy of Sciences, Flemingovo nám. 2, 16610 Prague, Czech Republic

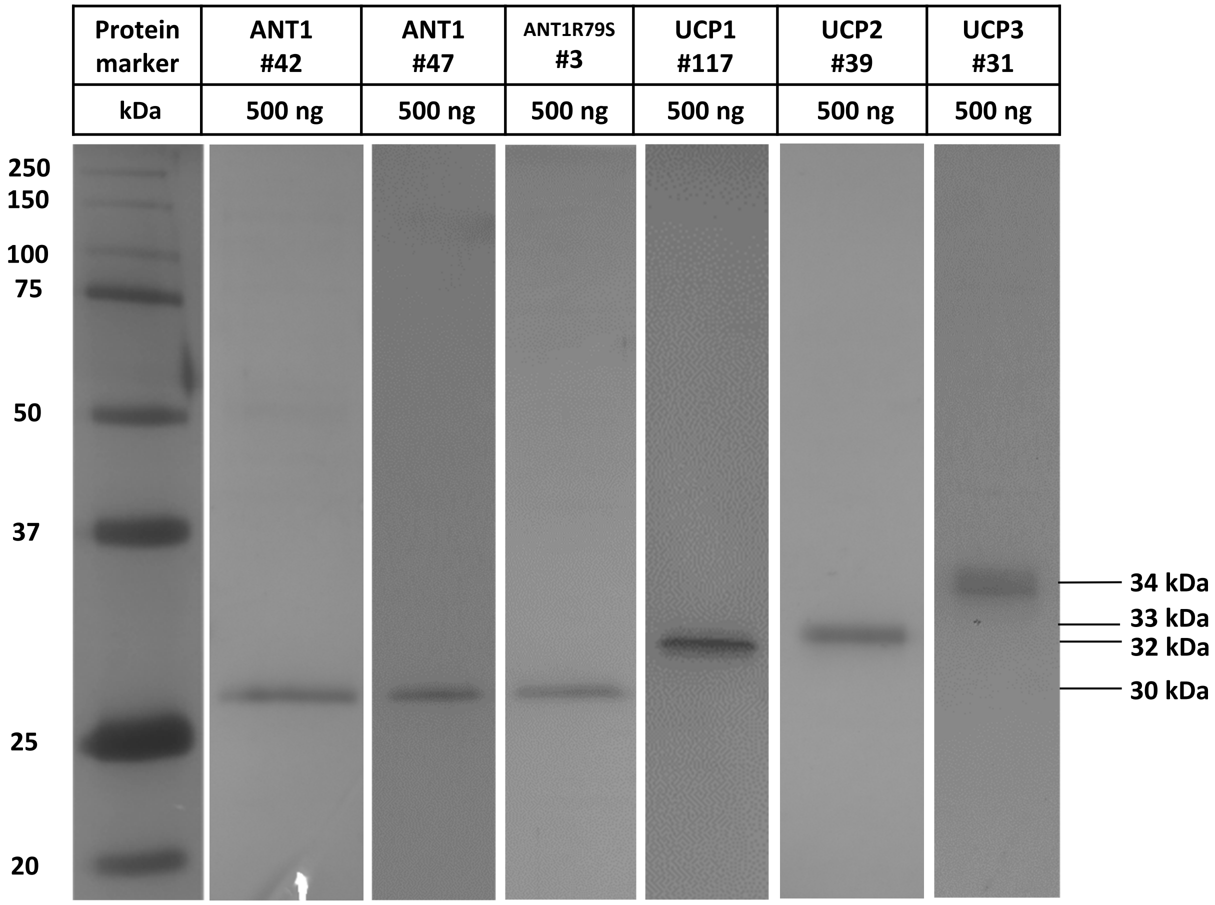

Figure S1. Representative silver staining of proteins used in the study. For the protein purity control, 500 ng of proteoliposomes were loaded onto 15% acrylamide gels and SDS-PAGE was conducted. Subsequently, proteins were visualized by silver staining. Precision Plus Protein Dual Color Standard was loaded as a molecular weight marker. Lanes depicted here are cut out from several independent gels. Full images are available at the following link: 10.5281/zenodo.5113039.

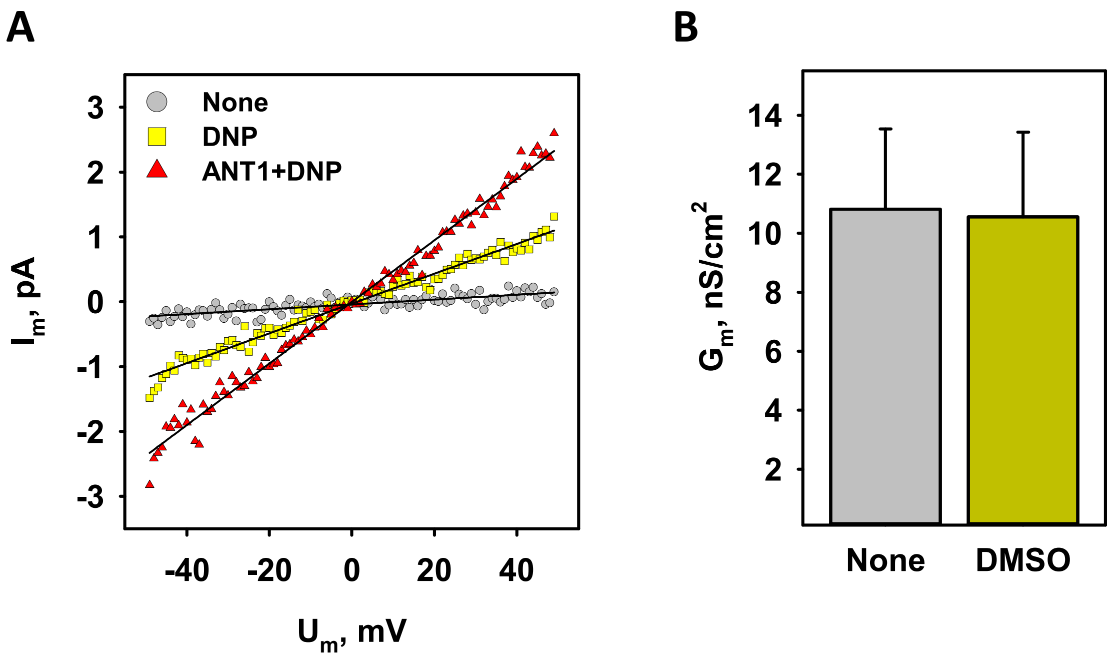

Figure S2. Representative current-voltage recordings of pure lipid bilayer membranes (grey circles) and membranes reconstituted with ANT1 (red triangles) or without ANT1 (yellow squares) in the presence of DNP (**A**). Membrane was made of DOPC:DOPE:CL (45:45:10 mol %). Lipid and protein concentrations were 1.5 mg/ml and 4 µg/(mg of lipid). Buffer solution consisted of 50 mM Na_2SO_4, 10 mM Tris, 10 mM MES and 0.6 mM EGTA at pH = 7.34 and T = 32°C.

DNP was dissolved in DMSO, which doesn´t alter the membrane conductance (**B**). Data are the mean ± SD of at least three independent experiments.

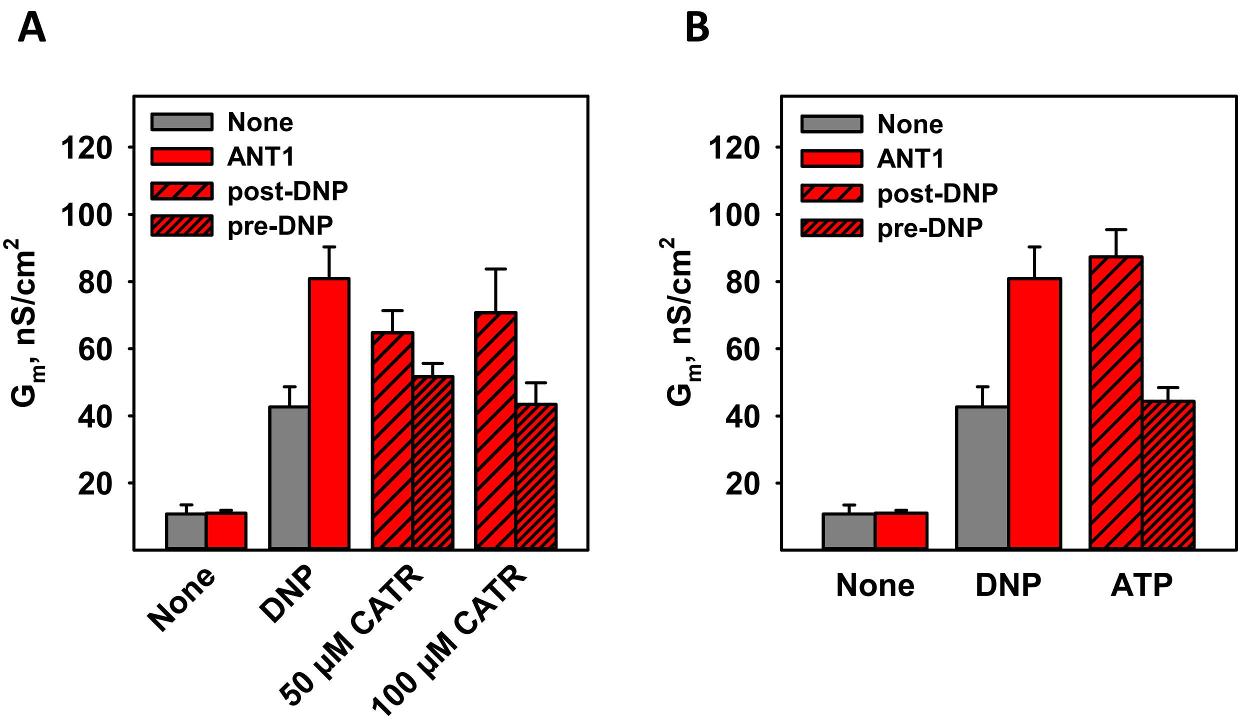

Figure S3. Effect of ANT1 specific inhibitors on the total conductance (G_m) of membranes reconstituted with ANT1 and DNP.

A. Increase of G_m in the presence of 50 μM DNP without ANT1 (grey) or with ANT1 (red). 50 or 100 μM CATR was added to the membranes after DNP (medium pattern) or before DNP (fine pattern).

B. G_m without ANT1 (grey) or with ANT1 (red) in the membrane in the presence of 50 μM DNP or 4 mM ATP. ATP was added to the membranes after DNP (medium pattern) or before DNP (fine pattern). Experimental conditions were similar to Fig. 2.

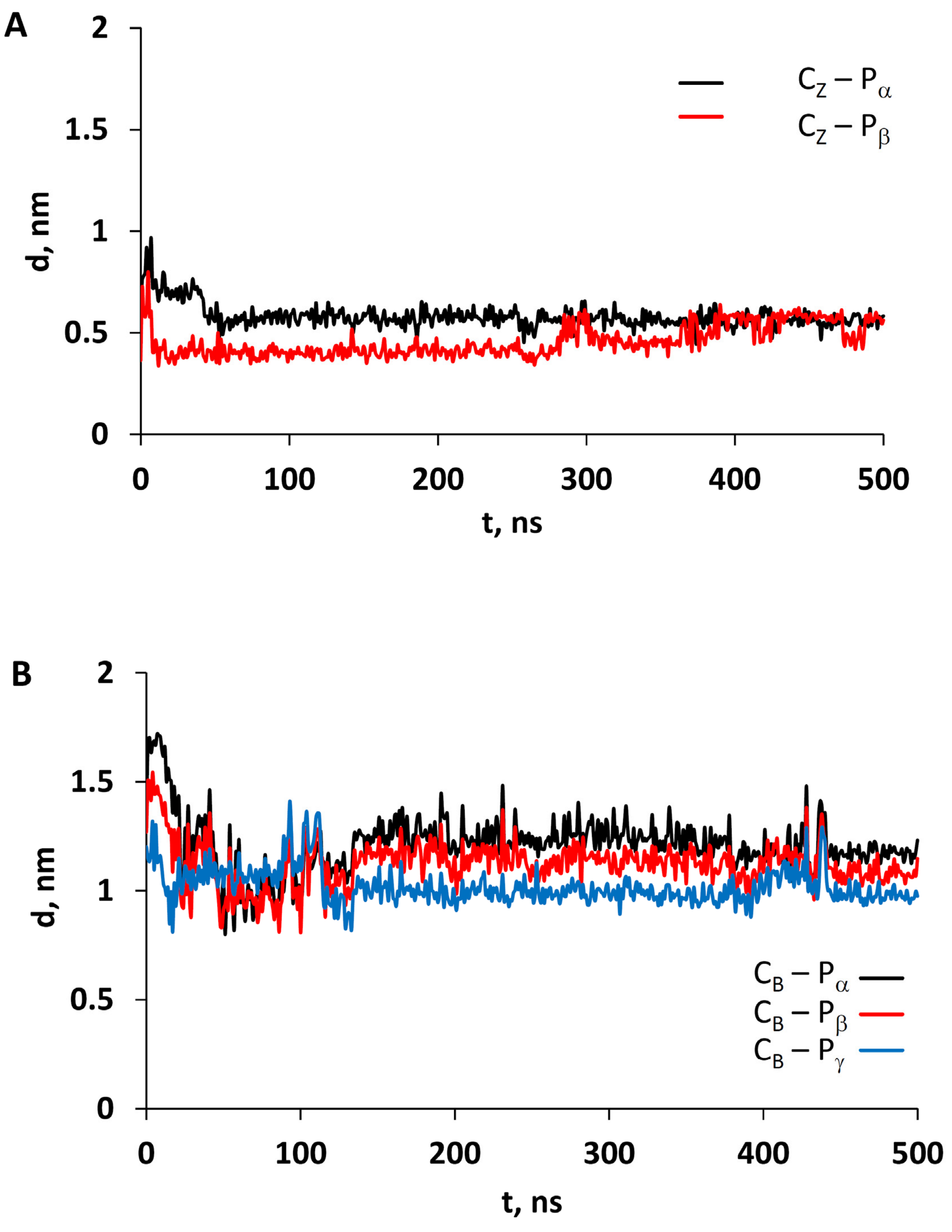

Figure S4. ATP binding to the arginine 79 of the ANT1 or ANT1-R79S.
Analysis of distances between phosphorous atoms in phosphate groups (P_α, P_β, and P_γ) and C_Z atom in R79 (**A**) and C_B atom in S79 residue (**B**), respectively. C_Z is central carbon atom of the guanidinium side chain in R79. C_B is carbon atom in the side chain of S79. Binding of P_γ to C_B atom occurs at larger distances and is omitted for clarity. MD simulations were performed for 500 ns.

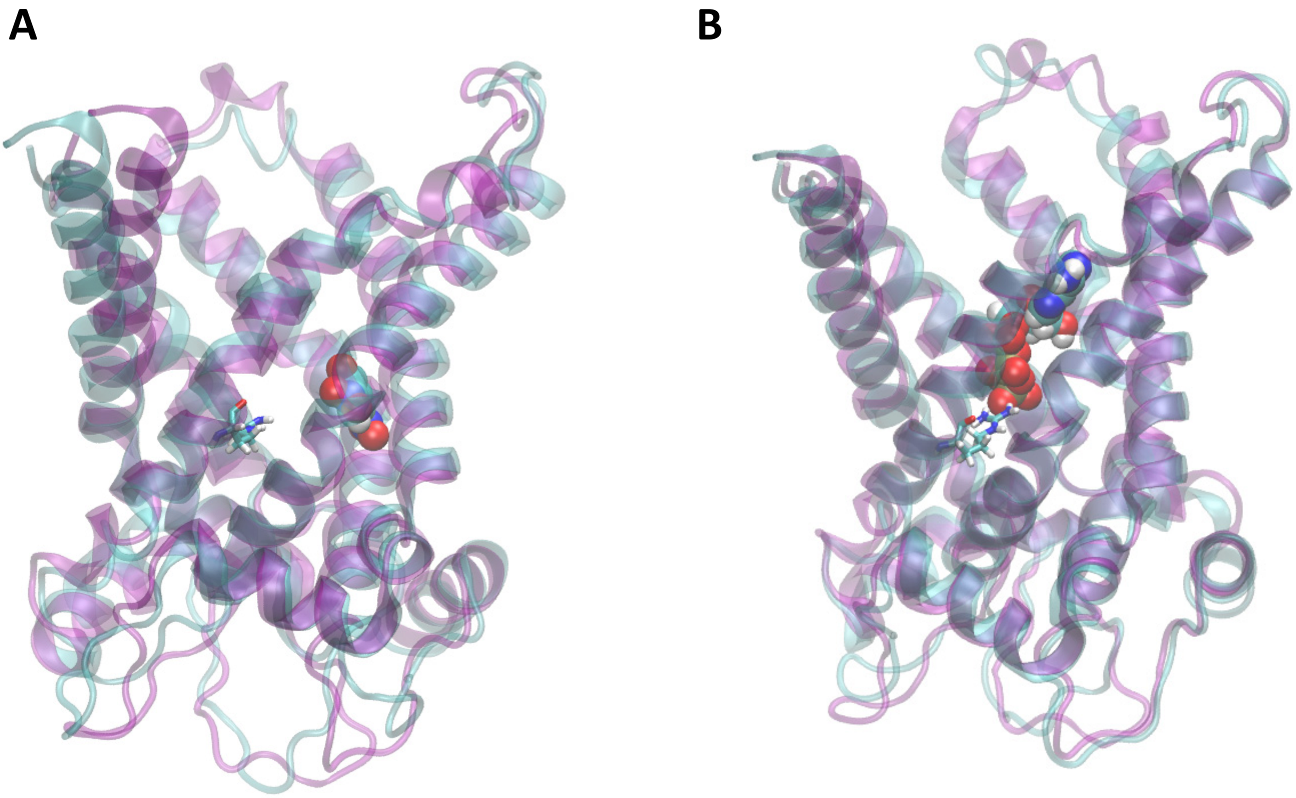

Figure S5. DNP and ATP binding in ANT1.

Alignment of the starting ANT1 structure (purple) with the ANT1 structure with bound DNP (**A,** cyan) and ATP (**B,** cyan) after 500 ns of MD simulations. Starting ANT1 structure was taken from Ref. 56. DNP and ATP are shown in vdW representation, R79 residue - in licorice.
